# Nrf2—A Molecular Target for Sepsis Patients in Critical Care

**DOI:** 10.3390/biom10121688

**Published:** 2020-12-17

**Authors:** Sandra Gunne, Ulrike Heinicke, Michael J. Parnham, Volker Laux, Kai Zacharowski, Andreas von Knethen

**Affiliations:** 1 Fraunhofer Institute for Translational Medicine and Pharmacology ITMP, Theodor-Stern-Kai 7, 60596 Frankfurt, Germany; sandra.gunne@ime.fraunhofer.de (S.G.); michael.parnham@ime.fraunhofer.de (M.J.P.); volker.laux@ime.fraunhofer.de (V.L.); 2 Department of Anaesthesiology, Intensive Care Medicine and Pain Therapy, University Hospital Frankfurt, Theodor-Stern-Kai 7, 60590 Frankfurt, Germany; ulrike.heinicke@kgu.de (U.H.); kai.zacharowski@kgu.de (K.Z.)

**Keywords:** sepsis, inflammation, resolution, detoxification, antioxidant defense

## Abstract

The transcription factor NF-E2 p45-related factor 2 (Nrf2) is an established master regulator of the anti-oxidative and detoxifying cellular response. Thus, a role in inflammatory diseases associated with the generation of large amounts of reactive oxygen species (ROS) seems obvious. In line with this, data obtained in cell culture experiments and preclinical settings have shown that Nrf2 is important in regulating target genes that are necessary to ensure cellular redox balance. Additionally, Nrf2 is involved in the induction of phase II drug metabolizing enzymes, which are important both in degrading and converting drugs into active forms, and into putative carcinogens. Therefore, Nrf2 has also been implicated in tumorigenesis. This must be kept in mind when new therapy approaches are planned for the treatment of sepsis. Therefore, this review highlights the function of Nrf2 in sepsis with a special focus on the translation of rodent-based results into sepsis patients in the intensive care unit (ICU).

## 1. Introduction

**Figure 1:**
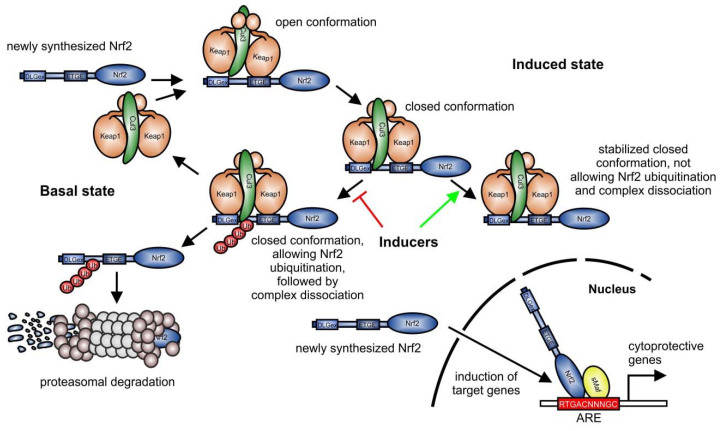
Keap1-dependent degradation of Nrf2 (modified from [18]). In the current concept [19], Keap1 links newly synthesized Nrf2 to proteasomal degradation via the cullin (Cul) 3-RING-box protein 1 ubiquitin ligase complex in the basal state. In an open conformation, Keap1 homodimers bind primarily to the ETGE-motif (amino acids 79-82), which promotes binding of the second Keap1 protein to the Nrf2 DLGex (amino acids 1–51) motif, associated with a closed conformation. Then, Cul3 can transfer ubiquitin residues to target Nrf2 for degradation by the proteasome. Following Nrf2 degradation, the Keap1/Cul3-complex is released and can target further Nrf2 proteins for proteasomal degradation. Inducers of Nrf2 stabilize the binding of the Keap1 homodimer to Nrf2, favoring a Keap1-Cul3-Nrf2 complex, sustaining Keap1, and not allowing Nrf2 ubiquitination, which is blocked in the induced state. Consequently, newly synthesized Nrf2 is not degraded and can induce the expression of target genes. Red T-bar = blocking Nrf2 degradation pathway; green arrow = favoring a stabilized complex, not allowing Nrf2 ubiquitination, and consequently preventing complex dissociation; ARE, antioxidant-responsive element.

Based on its role as the master regulator of the cellular antioxidant response, NF-E2 p45-related factor 2 (Nrf2) is a physiologically important transcription factor which belongs to the Cap ‘n’ collar transcription factor family [1,2]. It is ubiquitously expressed [3] and activated in the cell either by release from its binding to Kelch-like ECH-associated protein 1 (Keap1) [4], which is required to target Nrf2 for proteasomal degradation (Figure 1), or following new Nrf2 translation without degradation. Nrf2 then induces the expression of genes of the phase II family of drug metabolizing enzymes [5,6] and of antioxidant factors [7]. Keap1 inhibition is mediated by reactive oxygen species (ROS) or electrophiles [8,9], which oxidize the three main Keap1 cysteine residues needed for Nrf2 binding [10], thereby stabilizing Nrf2. Keap1 can also be targeted by p62/sequestosome1 (SQSTM1), an autophagy transport protein, which contains a Keap1 binding motif, thereby competing for interaction with Nrf2 [11,12]. Keap1 association with p62/SQSTM1 leads to autophagosomal degradation of the endogenous Nrf2 inhibitor, subsequently activating Nrf2. Whereas Keap1 links Nrf2 to the cullin (CUL) 3-RING-box protein (RBX) 1 ubiquitin ligase complex [13,14], the ß-transducin repeat-containing protein (ß-TrCP) links Nrf2 to S-phase kinase-associated protein-1 (SKP1)-CUL1-RBX1-dependent ubiquitination [15]. The latter process is enhanced by glycogen synthase kinase (GSK) 3-conferred phosphorylation of Nrf2 at its Nrf2-ECH homology (Neh) 6 domain, thereby stabilizing Nrf2-binding to ß-TrCP [16]. In addition to degradation-mediated Nrf2 regulation, posttranslational modifications (PTM) can also directly stabilize Nrf2. Among others, phosphorylation of serine 40 in Nrf2 by protein kinase C (PKC) has been shown to prevent its association with Keap1, thereby allowing Nrf2 to bind to antioxidant-responsive elements (ARE) in the promoter/enhancer regions of target genes [17].

Small musculo-aponeurotic fibrosarcoma (sMaf) proteins function as Nrf2 heterodimerization partners to improve binding of Nrf2 to DNA [20]. In addition to its regulation by proteasomal or autophagosomal decay, Nrf2-dependent gene induction can also be inhibited by the transcriptional repressor BTB domain and CNC homolog 1 (Bach1). This repressor competes with Nrf2 and sMaf proteins for binding to the ARE of enhancer regions of Nrf2 target genes, such as heme-oxygenase (HO)-1 [21] and nicotinamide adenine dinucleotide phosphate (NADPH) quinone oxidoreductase (NQO) 1 [22].

Consequently, the role of Nrf2 is most significant in diseases associated with the generation of large amounts of reactive oxygen species (ROS), leading to tissue and organ damage. One of these is sepsis. It is a major cause of fatal casualties in intensive care units (ICUs) worldwide [23] and is characterized by organ failure originating from a severe infection [24]. This evolves from an initial insult, consequently activating an immune response in the patient. In contrast to the normal situation, where the immune system tightly regulates pro- and anti-inflammatory responses, in sepsis progression, these mutually balancing mechanisms are uncoupled. Thus, a first hyper-inflammatory phase leads to the excessive expression of pro-inflammatory cytokines and the pronounced generation of reactive molecules such as ROS [25]. Accordingly, a hyper-inflammatory response is initiated. Interestingly, almost in parallel, an anti-inflammatory response is raised to counterbalance this hyper-inflammation. In association with T cell depletion, which compromises the patient’s immune system, this anti-inflammatory stage is often linked to the predisposition of the patient to a recurrent first infection or to a new secondary infection, which frequently is nosocomial [26]. Since imbalanced pro- and anti-inflammatory mechanisms may occur simultaneously, persistent inflammation and progressive immunosuppression are often associated with a bad outcome [27]. Therefore, a role of Nrf2 as a target for a new therapeutic concept in sepsis treatment is feasible.

## 2. Nrf2—Protection vs. Carcinogenesis

**Figure 2:**
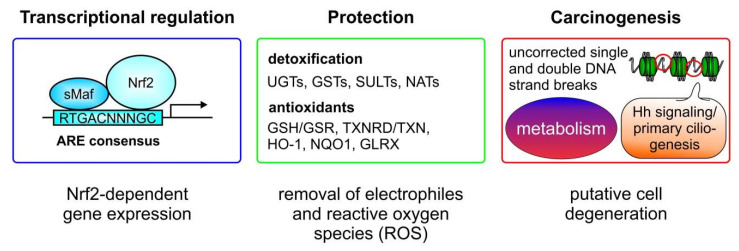
Nrf2—protection vs. carcinogenesis. Nrf2 binds as a heterodimer in combination with small musculo-aponeurotic fibrosarcoma (sMaf) proteins to antioxidant responsive elements (AREs) in the enhancer regions of promoters of Nrf2 target genes. Factors involved in the detoxification of electrophiles and xenobiotics, as so-called phase II enzymes, include UDP glucosylases (UGTs), glutathione-S-transferases (GSTs), sulfotransferases (SULTs), and arylamine-N-acetyltransferases (NATs). Proteins induced by Nrf2, that are required as antioxidant mediators to lower the reactive oxygen species (ROS) content, are the glutathione-reductase (GSR), regenerating the glutathione pool by reducing GSSG, and the thioredoxin reductase (TXNRD) which reduces oxidized thioredoxin (TRX). In relation to antioxidant capacity, heme oxygenase-1 (HO-1) contributes to heme and iron metabolism, the nicotinamide adenine dinucleotide phosphate (NADPH) quinone oxidohydrogenase 1 (NQO1) catalyzes the two- or four-electron reduction of endogenous and environmental quinones, and glutaredoxins—as small redox enzymes—reduce glutathione or non-glutathione disulfide substrates using GSH as a co-factor and electron donor. Finally, when excessive Nrf2 activity occurs, cells are protected against apoptosis, even following DNA damage which is not adequately corrected. These cells degenerate and can become the starting point for a tumorigenic process. Moreover, a metabolic change initiated by Nrf2 activation can promote tumorigenesis as well. Nrf2-dependent altering of hedgehog (Hh) signaling and primary ciliogenesis can also cause tumor initiation.

The transcription factor Nrf2 is the master transcriptional regulator of genes involved in cellular detoxification and antioxidant defense [7,28]. Nrf2 can hetero-dimerize with proteins of the sMaf family (Figure 2, left panel), binding collectively to DNA motifs known as antioxidant response elements (AREs). These contain the consensus nucleotide sequence 5′-RTGACNNNGC-3′. This regulatory element can be found in the enhancer regions of Nrf2 target genes, which facilitates its Nrf2-dependent induction [28].

Nrf2-induced genes are involved in the detoxification of electrophile compounds and xenobiotics. Genes encoding enzymes for detoxification include UDP-glucuronosyltransferases (UGTs) [29], glutathione-S-transferases (GSTs) [30], sulfotransferases (SULTs) [31], and arylamine- N-acetyltransferases (NATs) (Figure 2, middle panel) [32]. A second group of Nrf2-target genes are factors with antioxidant capacity, known to be important during inflammation-associated ROS production by the cells. Representatives of this group are responsible for regenerating the glutathione pool, and include glutathione-reductase (GSR), known to reduce oxidized GSSG back to reduced GSH [33] or to catalyze the first step of the *de-novo* synthesis of GSH, via the glutamate cysteine ligase (GCL), which consists of a catalytic (GCLC) and a modifier (GCLM) subunit [34]. Moreover, thioredoxin reductase (TRXR) is linked to Nrf2-dependent antioxidant defense by reducing oxidized thioredoxins (TRX). Additionally, HO-1 [2] and NQO1 [22] are also significant antioxidant mediators, whose expression is upregulated in response to Nrf2 activation. Enzymes directly involved in detoxifying ROS, such as superoxide anion (O_2_^−^), via superoxide-dismutase (SOD) [35] or hydrogen peroxide (H_2_O_2_) by catalase (CAT) activity [36], are also Nrf2 targets (their genes are).

Although very important in maintaining the cellular redox balance to protect cells against oxidative stress, Nrf2 can also be involved in carcinogenesis (Figure 2, right panel). On the one hand, augmented Nrf2 signaling can inappropriately prevent apoptosis, which allows the occurrence of mutations as starting points for cell degeneration. On the other hand, it has been shown that Nrf2 can be activated by a metabolite of the tricarboxylic acid cycle (TCA), also known as the citric acid cycle (CAC), such as itaconate [37]. Thus, recent evidence (reviewed recently in [38]) supports the notion that Nrf2-dependent modulation of cell metabolism can support cancer progression. Nrf2 can contribute to the regulation of cell metabolism signaling, such as the pentose phosphate pathway, NADPH generation, and lipid and amino acid turnover, which can be associated with carcinogenesis and tumor progression. Moreover, several studies have recently shown a connection between Nrf2 hyperactivation and primary cilia, which act as a tumor suppressor organelles (recently reviewed in [39]). Nrf2 hyperactivation simultaneously blocks Hedgehog (Hh) signaling by transcriptionally upregulating Patched 1 (PTCH1), as well as inhibiting primary ciliogenesis by augmenting p62/SQSTM1 expression [40]. PTCH1 is induced via Nrf2 binding to a newly identified ARE, whereas p62/SQSTM1 is an established Nrf2-target gene. PTCH1 negatively regulates Hh signaling and the autophagy adapter p62/SQSTM1 retains proteins which are important for primary ciliogenesis, such as oral-facial-digital syndrome 1 (OFD1) and Bardet–Biedl syndrome 4 (BBS4) in the cytosol [40]. Interestingly, another study found reduced primary ciliogenesis and Hh signaling in NRF2-null cells, mainly attributed to ARE sites, identified in the glioma-associated oncogene (GLI)2 and GLI3 genes, which are mandatory for cilia formation [41].

Although not a topic of this review, these possible consequences of Nrf2 activation should be born in mind when Nrf2 activation is considered as a therapeutic concept, especially for chronic therapies.

## 3. Preclinical Sepsis Models of Nrf2 Function

**Figure 3:**
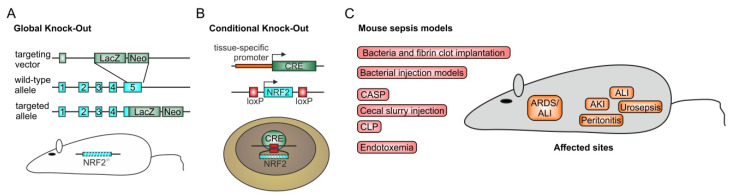
Nrf2 in murine sepsis models. To define the role of Nrf2 in sepsis, mouse models are recommended, as they allow deletion of functional Nrf2 expression completely (**A**) by a global knock-out [77], or tissue-specifically (**B**) as a so-called conditional knock-out with a floxed Nrf2 gene and the use of a deleter Cre mouse [42,44]. To mimic the sepsis condition of a patient in the intensive care unit (ICU), different mouse methods have been applied (**C**). Initial studies used bacterially contaminated fibrin clots, implanted into the peritoneal cavity to induce sepsis [78,79]. Simplified methods include immediate bacterial injection models which, contingent on the route of application, provoke peritonitis after intra-peritoneal injection [80,81] or urosepsis when injected into the bladder [82]. Moreover, inhalation or direct intra-tracheal inoculation of bacteria such as *Streptococcus pneumoniae* induces acute lung injury in its most severe form, acute respiratory distress syndrome (ARDS) [83,84]. ARDS and acute lung injury can also occur indirectly in response to polymicrobial sepsis initiation, as in colon ascendens stent peritonitis (CASP) [85,86], cecal ligation and puncture (CLP) [60,61]—the gold standard for mouse sepsis models—or cecal slurry injection [87]. All three models first induce peritonitis, so that the widespread infection, leads to a polymicrobial sepsis which often is associated with organ damage, such as acute kidney (AKI) [88] or liver injury [89], finally ending in multi-organ dysfunction (MODS) and death. Sometimes, endotoxemia is used. In this setting, lipopolysaccharide (LPS) from Gram-negative bacteria is injected intra-peritoneally or via the tail vein [90]. However, this model has been questioned as not really inducing sepsis, although it can cause a poor outcome, depending on the concentration of LPS used [91]. CDDO-Im-dependent Nrf2 activation has also been shown to improve endotoxemia survival [50].

Besides cell culture-based experiments, in vivo animal approaches are most important to studying, clarifying, and understanding the role of Nrf2. Due to the availability of gene knock-out animals, the role of Nrf2-dependent regulation of specific genes, which also can be used as read outs for functional Nrf2 expression, can be identified. Thus, mice have been generated with global (Figure 3A) or tissue-specific Nrf2 knock-outs (Figure 3B) [42,43]. Knock-out mice for the Nrf2 regulator Keap1 are also available [44]. Tissue specificity is accomplished with the use of Cre deleter mice [45,46], expressing the Cre recombinase in the desired tissue or cell type and crossing these mice with those containing a floxed Nrf2 gene (Nrf2^fl/fl^) [42]. In sepsis models, several settings have been established to mimic the sepsis patient. Different methods are available to induce sepsis in the mouse (Figure 3). Endotoxemia, involving the administration of bacterial lipopolysaccharide (LPS), leads to an early stage of sepsis called “systemic inflammatory response syndrome” (SIRS) in the absence of living pathogens and is used to specifically induce a toll-like-receptor-(TLR-)4-directed response of the murine immune system [47]. Depending on the LPS-concentration used, endotoxemia can also be associated with a poor outcome. LPS inhibits glutathione synthesis by attenuating sumoylation of Nrf2 and the heterodimerization partner MafG [48]. Thus, glutathione synthesis is reduced, which is consequently associated with increased vulnerability to ROS-dependent damage, leading to organ dysfunction [34]. Accordingly, counteracting Nrf2 inhibition by overexpressing the ubiquitin-conjugating enzyme 9 (Ubc9) [48], necessary for Nrf2 sumoylation, or directly activating Nrf2 by antioxidant compounds, such as the flavonoid dihydroquercetin, has been shown to protect mice against inflammation and to improve mouse survival [49]. This is also true for the potent small-molecule activator 1-[2-cyano-3-,12-dioxooleana-1,9(11)-dien-28-oyl]imidazole (CDDO-Im) [50]. In line with this, several other reports provide evidence for a protective role of Nrf2 in endotoxemia in mice or rats, among others, by inducing the Nrf2 target gene *HO-1* [51,52,53,54,55,56,57,58,59].

As outlined above, endotoxemia is also linked to organ damage. However, these disease-associated consequences are most pronounced in the more advanced mouse sepsis models, e.g., cecal ligation and puncture (CLP). In this setting, primarily peritonitis is induced, leading to multi-microbial sepsis [60,61]. Nrf2-knock-out mice have been used to determine the role of Nrf2 in this model [43,62]. The studies showed that increased inflammation is linked to a worse outcome in Nrf2 knock-out mice compared to control animals. Accordingly, knocking out Keap1, the physiological regulator for endogenous Nrf2 level, reduced the inflammatory response and ameliorated organ damage and survival of CLP-treated mice [44]. Furthermore, in the CLP-model, the use of putative antioxidants such as resveratrol [63], mangiferin [64], artesunate [65], myricetin [58], amentoflavone [66], dimethyl fumarate [67,68], and ascorbic acid [69] mediated Nrf2 activation, which reduced organ damage and improved rodent survival.

Organs which are often affected during sepsis progression are the kidney, liver, heart, and lung. Polymicrobial sepsis can initiate acute kidney injury (AKI), characterized by the release of kidney damage markers, such as creatinine and lipocalin-2 (Lcn-2), into the serum [70,71]. Consistent with the need for high endogenous antioxidant capacity, activation of Nrf2 has been shown to prevent or at least significantly reduce AKI in this disease model [64,72,73,74]. Similarly, sepsis-initiated acute liver injury can be prevented or ameliorated by pharmacological Nrf2 activation or by its genetic stabilization by Keap1 disruption [44,75]. This also accounts for acute lung injury, and its most severe occurrence is acute respiratory distress syndrome (ARDS). Nrf2-dependent gene induction rescues this sepsis-dependent organ damage that is mainly caused by an overwhelming production of ROS [76].

**Figure 4:**
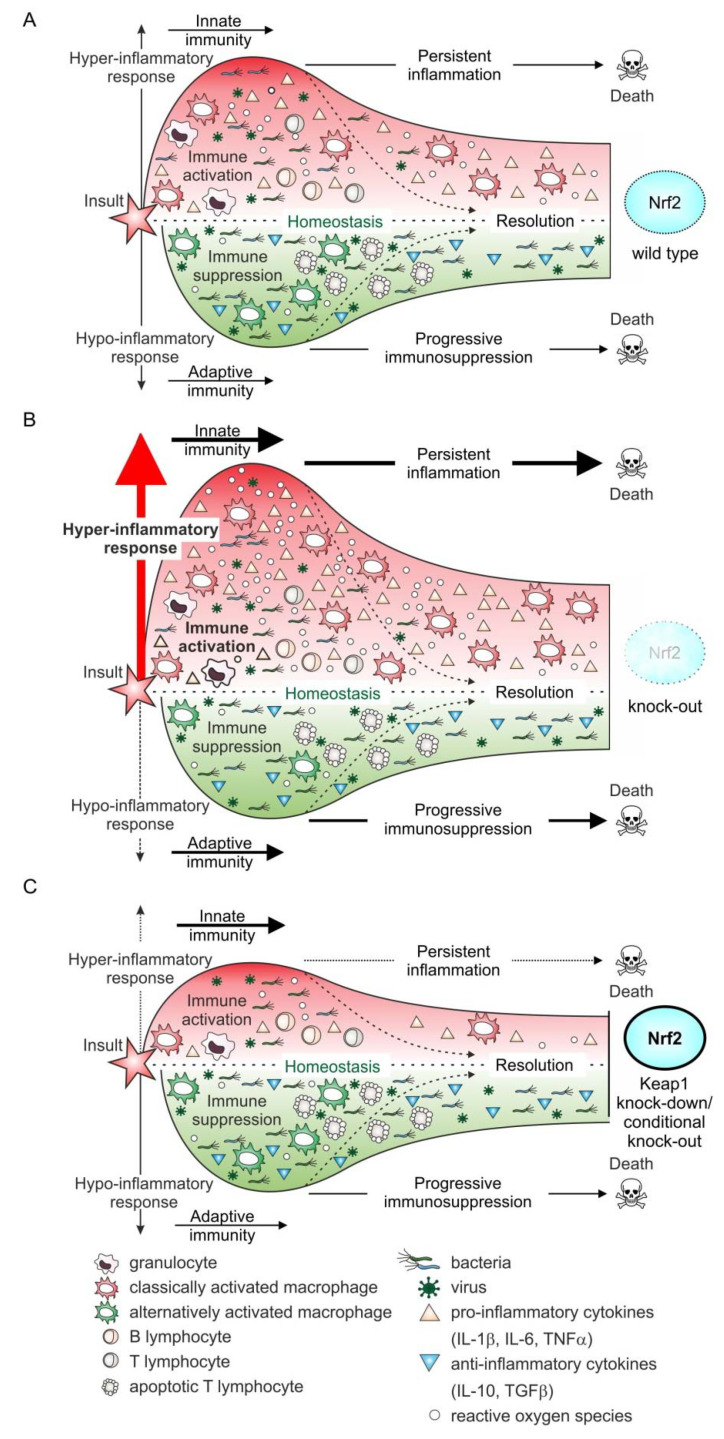
Nrf2 and the immune response during sepsis progression. In the Nrf2 wild type situation (**A**), the immune system is activated due to an initial insult. This leads to the activation of cells of the innate response, such as granulocytes and monocytes/macrophages. The latter are classically activated following bacterial or viral exposure, a process enhanced by IFNγ, released from recruited helper T lymphocytes (CD4^+^), which are adaptive immune cell. The adaptive immune response is characterized by excessive expression of pro-inflammatory cytokines and the massive release of reactive oxygen species [92,93]. During sepsis’ development and progression, this phase can persist, conferring permanent inflammation and preventing its resolution, resulting in a poor outcome. Almost in parallel, an anti-inflammatory response is initiated. This is distinguished by the expression of anti-inflammatory cytokines, such as IL-10, and the phenotype switching of macrophages to the alternatively activated phenotype. Moreover, T cell apoptosis and consequently T cell depletion are accompanied by an inappropriate immune response towards the initial or a second new infection, which is also associated with decreased survival rate. Although Nrf2 is stabilized, especially following the overwhelming production of pro-inflammatory mediators, its activation and the resulting expression of Nrf2-dependent target genes is not sufficient to cope with the excessive oxidative stress. Thus, the redox balance is significantly destabilized. This is further enhanced when functional Nrf2 expression is deleted (**B**). In this case (knock-out, loss of function mutation) [98,99], the hyper-inflammatory response is considerably intensified, and consistently associated with increased tissue and organ damage, provoking a worsened outcome. In line with this observation, reverting to functional expression of Nrf2 or increasing it by inhibition/deletion of the main Nrf2 destabilizer, Keap1, will reduce the hyper-inflammatory phase, consequently improving the outcome with sepsis (**C**). This can be achieved genetically (knock-out by CRISPR/Cas) [96,97] or pharmacologically by stabilizing Nrf2, e.g., after modulation by small molecules [100,101].

In Nrf2 wild type animals (Figure 4A), the immune system in sepsis is activated by some initial trauma, which primarily leads to the expression of pro-inflammatory cytokines and the generation of inflammation-promoting mediators such as ROS. When excessively generated systemically, this can cause a hyper-inflammatory response, favoring a poor outcome. Thus, in this condition, macrophages are mainly classically activated (M1 type). Nevertheless, an anti-inflammatory response is initiated almost in parallel, which is characterized by the alternative activation of macrophages (M2 type) and T cell apoptosis, finally provoking immune suppression [92,93]. As an appropriate response to the recurrent infection or a newly occurring second infection cannot be effectively mounted, the hypo-inflammatory state is often associated with organ damage and death. Under normal conditions, inflammation is terminated endogenously, leading to the resolution of inflammation. However, persistent inflammation or progressive immunosuppression can prevent this. With regard to the role of Nrf2, especially in the resolution of inflammation, its state-specific activation to reduce the hyper-inflammatory response or promote a hypo-inflammatory response might be advantageous. The former can be achieved by fostering Nrf2–Keap1 stabilization to prevent ROS-mediated activation of pro-inflammatory gene expression or ROS-dependent cell and tissue damage. A hypo-inflammatory state could be overcome by the inhibition of Nrf2, maintaining a pro-inflammatory status. Adapted fine tuning would be necessary to cope with the relevant reaction. The lack of the functional expression of this important transcription factor, as in the global Nrf2 knock-out model, renders the system prone to an enhanced hyper-inflammatory response, compared to the Nrf2 wild type immune response during sepsis progression (Figure 4B) [43,62]. This is closely associated with increased cell and tissue damage, consequently leading to multi-organ dysfunction (MODS) and death. Interestingly, the Keap1 null mouse is postnatally lethal. Most likely, this is caused by malnutrition resulting from hyperkeratosis in the esophagus and forestomach [94]. Therefore, only tissue specific, so-called conditional Keap1 knock-out mice are feasible (Figure 4C)—among others, mice lacking functional Keap1 expression in immune cells of the myeloid lineage (LysM-Cre Keap1^−/−^) [44] or in hepatocytes (Alb-Cre Keap1^−/−^) [44,95]. Mice with a Keap1 deletion in the myeloid lineage confer significantly reduced sepsis progression and improved survival [44]. In mice with a hepatocyte-specific Keap1 knock-out, liver damage in response to T cell–mediated acute inflammatory liver injury is significantly decreased, as determined by less serum alanine aminotransferase (ALT) released as a liver damage marker [95]. These Keap1 knock-out studies showed a similar protective effect as that with Nrf2 wild type mice treated with Nrf2 stabilizing compounds. The recently discovered CRISPR/Cas9 technology has translated the Keap1-knock-out approach to human cells. Thus, CRISPR/Cas9-mediated gene targeting was used to generate a homozygous Keap1-knock-out human embryonic stem cell line [96], and transient CRISPR/Cas9-dependent Keap1 editing for therapeutic Nrf2 activation in primary human T lymphocytes [97]. 

## 4. Nrf2 in Immune Cell Subpopulations

In light of these differences in the immune response when Nrf2 is deleted or activated, it is important to determine the impacts of Nrf2 on different immune cell subpopulations. Thus, various studies have analyzed the roles of Nrf2 in lymphocytes, granulocytes, dendritic cells (DCs), and monocytes/macrophages (Table 1). 

### 4.1. B Lymphocytes

Terminal differentiation of murine B lymphocytes to antibody-producing plasma cells is associated with endogenous H_2_O_2_ production. It has been shown that B-cell receptor (BCR) signaling is fostered by H_2_O_2_ production in B cells, occurring in parallel [125]. Moreover, the expression of Nrf2-dependent genes has been summarized [126]. These observations are supported by the study of Bursley et al. [103]. Using LPS to T cell-independently activate B cells, derived from Nrf2 wild type or Nrf2 knock-out mice, subsequent stimulation with the Nrf2 activator tert-butylhydroquinone (tBHQ) caused significantly enhanced IgM production in Nrf2 wild type B cells. This was absent in Nrf2-deficient B lymphocytes. Considering the expression of the Nrf2 target gene HO-1 as an anti-apoptotic protein, it was investigated whether its expression is upregulated in neoplastic B cells. The α/β-unsaturated ketone electrophilic lipid 15d-PGJ_2_, a Michael acceptor agent, reacts in Michael additions with cysteine residues of proteins, including Keap1 [127]. Thus, 15d-PGJ_2_ provokes Nrf2 activation and was used to analyze the expression of HO-1 in murine and human normal and neoplastic B cells [104]. In this setting, the authors demonstrated that the capacity of malignant B cells to express HO-1 in response to 15d-PGJ_2_ stimulation was higher than in normal B cells. Nrf2-dependency was verified by Nrf2 siRNA experiments and the use of B cells derived from Nrf2 knock-out mice [104]. Recently, it has been proposed that electrophilic Michael acceptor-containing drugs may be useful in the treatment of inflammation and cancer [128,129]. In fact, reaction of nitric oxide and nitrite-derived species with polyunsaturated fatty acids yields electrophilic fatty acid nitroalkene derivatives (NO2-FA), which display anti-inflammatory properties, including inhibition of sepsis-induced pulmonary inflammation [130]. 

### 4.2. Dendritic Cells (DCs) 

Nrf2 is also expressed in dendritic cells (DCs), where its expression is responsible for redox balance [131]. In DCs exposed to glioma-conditioned medium, Nrf2 was found to inhibit the maturation of DCs and consequently to lower T cell-dependent immunity. This was shown by an siRNA approach directed against Nrf2, which significantly increased DC maturation and downstream T cell activation [105]. These results were confirmed in bone marrow-derived immature DCs (iDCs) of Nrf2 wild type and knock-out mice. In Nrf2-defcient iDCs, the basal GSH level is lower compared to Nrf2-expressing DCs [106], which allows upregulation of co-stimulatory proteins in Nrf2-negative DCs. Moreover, in these cells, phagocytic functions are reduced, but antigen presentation and linked T cell activation are increased [106]. As Nrf2-deficient mice have been shown to be more susceptible to severe lung inflammation, associated with the expression of Th2 lymphocyte-derived cytokines such as IL-4 and IL-13 [132], Williams et al. [107] observed Nrf2-deficient DCs to be mediators of a Th2-mediated immune response. 

### 4.3. Granulocytes

In keeping with their role in the immune response, granulocytes produce large amounts of ROS to fight against pathogens. Due to the detrimental nature of ROS also towards the cells that generate them, granulocytes need a system to protect themselves against ROS. Therefore, Nrf2 is highly expressed in these cells [109]. To determine the role of Nrf2 in granulocytes, Chen et al. used a murine model of cardiac dysfunction in sepsis and endotoxemia [59]. In this setting, the authors identified the chemokine receptor CXCR2 as an Nrf2 target gene, responsible for neutrophil migration. Thus, interestingly, trimetazidine (TMZ)-dependent Nrf2 activation is associated with improved cardiac outcome due to increased neutrophil immigration. In addition, in the study of Helou et al., Nrf2 regulated CXCR2-dependent neutrophil migration, independently of the expression level of this receptor, which was similar in neutrophils derived from Nrf2 wild type and Nrf2-deficient mice [108], suggesting that the activation rather than the expression of the receptor is modified by Nrf2. Although Nrf2 is abundantly expressed in granulocytes [109], its myeloid expression is not necessary for wound healing in mice, indicating an alternative protective antioxidant system in these cells, such as glutathione peroxidase [133,134,135]. However, upon classical pro-inflammatory stimulation, e.g., by LPS, Nrf2 activation by triterpenoids such as CDDO-Im and CDDO-Me is protective in neutrophils [110]. Thus, neutrophils expressed Nrf2 target genes (GCLC, GCLM, NQO1, HO-1) associated with reduced ROS generation following LPS, fMLP, TNF-α, or 12-O-tetradecanoylphorbol-13-acetate (TPA) treatment. 

### 4.4. Monocytes/Macrophages

Together with granulocytes, monocytes/macrophages are innate immune cells representing the first line of defense towards pathogens. Although recent evidence supports the notion that monocytes/macrophages can acquire a kind of trained immunity, allowing improved host defense through metabolic and epigenetic changes (summarized in [136]), as cellular components of innate immunity, these mononuclear phagocytes are mainly dependent on unspecific cytotoxic mechanisms such as ROS generation by the NADPH oxidase Nox2 to kill pathogens. Therefore, like granulocytes, as described above, they need an antioxidant defense system, and consequently, Nrf2 is expressed in monocytes/macrophages. Unexpectedly, bone-marrow-derived Nrf2-deficient macrophages show no increased ROS production or alteration in co-stimulatory protein expression when activated. Nevertheless, antigen-driven CD8^+^ T cell functions are impaired [112]. Mechanistically, limited GSH and Cys availability leads to reduced T cell responses, pointing again to the antioxidant role of GSH and thiol groups. By analogy, Nrf2 activation following sulforaphane (SFN) treatment prevented HIV infection of human buffy coat-derived macrophages [111]. Although the mechanism has not been completely elucidated, Nrf2 activation prevented integration of the virus genome, following its reverse transcription into the host chromosomes. Besides synthetic compounds, known to stabilize Nrf2 by modifying Keap1, itaconate has been shown to alkylate cysteine residues of Keap1 [113]. Itaconate links cellular metabolism to Nrf2 activation. Following LPS-stimulation of macrophages, the immune-responsive gene 1 (Irg1) is induced, leading to the conversion of aconitate, a component of the tricarboxylic acid cycle, to its metabolite itaconate, inhibiting the succinate dehydrogenase (SDH) to modulate intracellular succinate levels [137]. Type I interferons enhance itaconate production by inducing Irg1 expression. In a negative feedback loop, itaconate activated Nrf2 to limit interferon type I expression and pro-inflammatory signaling [113,138]. Apart from this link to cell metabolism, cell-permeable derivatives of itaconate (dimethyl itaconate (DMI) and 4-octyl itaconate (OI)) have already been described to reduce lethality of endotoxemia in mice [113,139]. In line with this, protection following Nrf2 activation is also mediated by improving the phagocytic capability of alveolar macrophages derived from chronic obstructive pulmonary disease (COPD) patients by directly upregulating expression of the scavenger receptor, macrophage receptor with collagenous structure (MARCO) [114]. An Nrf2-dependency is further demonstrated by the finding in Nrf2-deficient mice, that SFN-dependent phagocytosis of bacteria by COPD alveolar macrophages is abolished [114]. Moreover, Nrf2 has been shown to act not only by inducing antioxidant target genes, but also to directly bind in the proximity of pro-inflammatory genes such as IL-6 and IL-1β, inhibiting recruitment of RNA polymerase II [115,140]. 

### 4.5. T Lymphocytes

Finally, for the second subpopulation of lymphocytes, the T cells, several studies have shown the involvement of Nrf2 in T cell differentiation and activation. A number of different T cell subpopulations exist. One of these belongs to the innate immune system, the so called invariant natural killer T cells (NKT). Although less is known about a Nrf2-dependency in these cells, experiments in mice with a CD4^+^ T cell specific deletion of Keap1 revealed disturbed development of NKT cells, causing a reduced number in mice. This is associated with higher rates of NKT cell proliferation and apoptosis, and increased mitochondrial activity and glucose uptake [116]. All functions were restored in mice in which Nrf2 was deleted in parallel.

Regulatory T cells (T_regs_), as a T cell subpopulation belonging to the adaptive T cell group, specifically express the transcription factor FoxP3 and are important in maintaining T cell tolerance, thereby preventing autoimmunity. It could be demonstrated that in old female Nrf2 knock-out mice a multi organ autoimmune syndrome occurred (Ma et al. 2006). To specifically analyze the role of Nrf2 in T_regs_, Klemm et al. used FoxP3-driven Keap1 knock-out mice [117]. However, considering the lethal outcome of global Keap1 knock-out mice, it was not surprising that FoxP3-driven, Keap1 knock-out mice died postnatally as well. This is due to the expression of FoxP3 not only in T_regs_ but also in epithelial cells, which probably accounts for death when Keap1 is deleted [141]. With these mice, the authors observed less FoxP3 T_regs_, consequently associated with reduced peripheral tolerance [117]. Employing sodium-butyrate (NaB) as an Nrf2-activating compound, Chen et al. [118] monitored a significant inhibition of the inflammatory response in murine experimental autoimmune uveitis (EAU). NaB administration reduced the count of Th17 cells and increased the number of T_regs_ in draining lymph nodes and spleens. Mechanistically, the differentiation of Th17 cells was attenuated by a Nrf2/HO-1 dependent mechanism. As a corollary, the blockade was revered by the HO-1 specific inhibitor tin protoporphyrin IX (SnPP). 

Th17 cells are an important T cell subpopulation linked with asthma and airway inflammation. These cells are known to secrete, among others, IL-17 [142]. In a study by Al-Harbi et al. [119], the authors demonstrated an increase in the total number of Th17 cells and reduced expression of IL-17A and IL-23 after SFN-dependent Nrf2 activation in a mouse model of mixed granulocyte airway inflammation. Mimicking graft versus host disease (GvHD) during allogenic hematopoietic transplantation (allo-HCT) in the mouse system showed ameliorated GvHD in donor CD4^+^ T cells with no functional expression of Nrf2 compared to allo-HCT from Nrf2 wild type mice [120]. This was associated with increased longevity of T_regs_ positive for Helios in allograft recipients, which may provide an explanation for reduced systemic inflammation following allo-HCT. In Nrf2-deficient donor CD8^+^ cells, cytotoxicity was not altered towards allogenic target cells. In order to translate a role of Nrf2 to human T cells, Noel et al. [97] used the human Jurkat T cell line and primary human T cells derived from peripheral blood. On editing KEAP1 with a CRISPR/Cas9 approach, expression of Nrf2 target genes (GCLM, NQO1, HO1) was significantly upregulated in Jurkat T cells and primary human T cells. Moreover, the number of CD4^+^ T cells increased, whereas the proportion of CD8^+^ T cells decreased in KEAP1-edited T cells. Interestingly, in enriched T_regs_, there was a shift to CD69^+^/IL-10^+^ T_regs_ compared to control T_regs_. The same group provided evidence, that T lymphocyte-specific, genetic amplification of Nrf2 by CD4-Keap1-KO, protected mice from ischemia-reperfusion (IR) and ameliorated AKI [121]. In these mice, the number of CD25^+^ Foxp3^+^ T_regs_ was increased. Enhanced Nrf2 reduced TNF-α, IFN-γ, and IL-17 expression in these cells. Additionally, adoptive transfer of T cells with a stabilized Nrf2, reduced AKI following IR also in the recipient animal [121]. This study supports the assumption that T-cell specific Nrf2 activation is protective towards acute injury. 

Early after T cell activation, Nrf2 is important to increase IL-2 expression and enhance c-Jun activation. In contrast, the expression of IFN-γ and TNF-α was reduced. This was shown in splenocytes derived from Nrf2 knock-out compared to wild type mice, using the two Nrf2 activators tBHQ and CDDO-Me [122]. Nrf2 activation in T cells seems to suppress Th1 differentiation, favoring a Th2 shift. A similar observation was made by Rockwell et al. [123]. In this study, tBHQ-dependent Nrf2 activation of negatively enriched CD3^+^ or CD4^+^ T cells derived from spleens and lymph nodes of Nrf2 knock-out and wild type mice were compared. The Nrf2 activating compound was added to freshly isolated splenocytes 30 min before classical T cell activation by αCD3/αCD28 antibodies. tBHQ concentration dose-dependently inhibited the expression of the Th1 cytokine IFN-γ in Nrf2 wild type mice, whereas it was significantly less effective in Nrf2-deficient cells. Induction of Nrf2 target genes (HO-1, NQO1, GCLC) was only observed in Nrf2 wild type T cells in response to tBHQ. Interestingly, tBHQ in Nrf2 wild type T cells upregulated the Th2 marker IL-4 as well. A similar increase was found for IL-5 and IL-13, which are also Th2 cytokines. In Nrf2-deficient T cells, tBHQ did not induce the expression of Th2 markers. These last two studies suggested a role of Nrf2 in mediating a Th2 shift. Finally, Morzadec et al. [124] provided evidence that in human T cells, obtained from buffy coats of healthy donors, expression of Nrf2 is induced and the Nrf2 protein is activated following αCD3/αCD28 activation. Consequently, Nrf2 target genes were induced. However, in contrast to murine T cells, Nrf2 did not alter expression of pro- or anti-inflammatory cytokines, suggesting different paths of activation in human vs. mouse T cells. 

Taken together, in mouse models, ex-vivo or in cell culture, Nrf2 activation, when appropriately performed, is an important tool for the development of therapeutic strategies for sepsis patients, since the function of Nrf2 is generally protective by downregulating pro-inflammatory signaling. 

## 5. Nrf2 in the Sepsis Patient

In “ClinicalTrials.gov”, 21 clinical trials are registered which involve Keap-1-dependent and Keap-1-independent approaches to Nrf2 activation and treatment of inflammation in patients. Furthermore, searching “ClinicalTrials.gov” for Nrf2-activating compounds identified 31 clinical trials for the synthetic triterpenoid CDDO-Me, 13 for RTA-408, 114 for di- and mono-methyl fumarate, 6 for oltipraz, 160 for ursodiol, 73 for SFN, 4 for sulforadex (SFX-01), 240 for the stilbene curcumin, 173 for the stilbene-derivate resveratrol, and finally, 11 for the nitro-fatty acid CXA-10 [143]. Summing up, this means roughly more than 800 clinical trials are focusing on Nrf2-dependent therapy concepts. Considering the organs that are mainly affected during sepsis, such as the lung (ARDS), liver (ALI), kidney (AKI), and heart (coronary artery disease/CAD), several clinical phase I, II, III, and IV trials elucidating the feasibility and functions of various Nrf2-activating compounds are already recruiting, running, or have been completed (for a review see [3]). As a common attribute, all of these activators provoke an electrophilic modification of KEAP1-Cys-151 as their mechanism of action, consequently activating Nrf2. CDDO-Me is used in clinical trials dealing with hepatic impairment, chronic kidney disease (CKD), and pulmonary hypertension (PH). Dimethyl fumarate (marketed as Tecfidera^®^ for multiple sclerosis) is being applied in rheumatoid arthritis and psoriasis, oltipraz to treat nonalcoholic steatohepatitis, the biliary acid ursodiol in chronic hepatitis C, SFN in COPD and asthma patients, curcumin in patients with CAD, AKI, and CKD, resveratrol for COPD and chronic subclinical inflammation, and CXA-10 to treat AKI and pulmonary arterial hypertension (PAH). As exemplified here, the use of Nrf2-activating compounds with respect to specific tissue or organ damage is already in clinical trials, which will, undoubtedly, end up with a licensed therapy concept, which putatively can be transferred to the sepsis patient. However, no clinical trials addressing the role of Nrf2 in sepsis patients are enrolled, as also outlined in a recent review [144]. 

Apart from Nrf2 activation, tools mimicking the function of Nrf2 are also already in clinical trials. Considering glutathione (GST) as the most widely available intracellular antioxidant peptide, the ratio of its oxidized form GSSG compared to the reduced GSH and its total amount are important markers for cellular redox potential. Due to this major role of GSH in fighting reactive oxygen species-dependent modifications, induction of its generator systems in response to Nrf2 activation, as described above, might be a therapeutic concept. Restoration of the intracellular glutathione pool by the glutathione precursor molecule N-acetyl cysteine (NAC) has already been described in animal experiments to reduce pro-inflammatory cytokine expression and to improve disease outcome [62,145] and administration of the synthetic GSH peroxidase mimic, ebselen, has broad anti-inflammatory activity in infectious and non-infectious conditions [146,147,148]. According to these preclinical data, NAC has also been used in the sepsis patient, however with controversial results [149,150,151,152,153]. In a prospective, randomized clinical trial, including 60 critical ill patients with sepsis in total, intermittent NAC infusion was compared with continuous infusion [149]. In this study, blood samples were collected to determine the total antioxidant capacity (TAC) and malondialdehyde (MDA) content. TAC assessment was performed before and after treatment and based on a commercially available assay analyzing the ability of antioxidants in serum or plasma samples to inhibit peroxidase-dependent oxidation of 2,2′-azino-bis(3-ethylbenzothiazoline-6-sulfonic acid) (ABTS). MDA is a byproduct of lipid peroxidation and thus, often used to determine oxidative stress. In this study, TAC was increased following intermittent and continuous treatment compared to the placebo group. The MDA level was decreased in both of the NAC-treated groups. However, there were no significant differences in TAC and MDA between the intermittent and the continuous treated groups. A study using high dose NAC treatment in mechanically ventilated multiple trauma critically ill patients, revealed a slightly higher mortality rate and an increase in ventilator days in the NAC-treated group, which were not significant [151]. Although NAC reduced oxidative stress, thereby also decreasing pro-inflammatory cytokine expression or the respiratory burst, this diminution could have been associated with poor clearance of pathogens [154]. Results obtained from studies using NAC as a putative mimic of Nrf2 activation, for instance, to reduce oxidative stress by augmenting the GSH-pool in sepsis patients, have to be taken into account, when Nrf2 is chosen as a direct target for setting up a new therapeutic approach. 

## 6. Conclusions

From these data, it is obvious that Nrf2 is a putative target, whose activation or inhibition can interfere with the hyper- or hypo-inflammatory response observed during sepsis progression. Therefore, its activation or inhibition should be monitored in initial clinical trials. Due to the likelihood of only using Nrf2 activating compounds acutely in sepsis treatment, the carcinogenic potential of constitutive Nrf2 activity is probably negligible.

Interestingly, recent progress in the understanding of COVID-19 infection and antioxidant strategies involving Nrf2 to prevent viral uptake, RNA- or DNA replication, and cytokine expression are the topics of current research [92,144,155], which possibly will give rise to new treatment strategies in sepsis.

## Figures and Tables

**Table 1:** Role of Nrf2 in the function of immune cell subpopulations. To characterize a putative role of Nrf2 in the treatment of sepsis patients, it is important to know how the activation of Nrf2 and its downstream signaling affect cell subpopulations of the immune response. In immune cells of the innate and adaptive immune systems, Nrf2 has been shown to be important for differentiation and function. Thus, B lymphocytes, dendritic cells, granulocytes, monocytes/macrophages, natural killer cells, and T lymphocytes express Nrf2 and are regulated by its signaling. ↑ upregulated, ↓ downregulated, ↓↑ not altered; RTA-402 (CDDO-Me, BARD-Me, bardoxolone methyl); RT-408 (omaveloxolone); SFN, sulforaphane; DMF, dimethyl fumarate; DEM, diethylmaleate; 15d-PGJ_2_, 15-deoxy-Δ^12,14^-prostaglandin J_2_; tBHQ, tert-butylhydroquinone; TMZ, tert-butylhydroquinone; NaB, sodium butyrate; ab, antibody; AKI, acute kidney injury; COPD, chronic obstructive pulmonary disease; IR, ischemia-reperfusion.

Immune Cell Subtype	Model	Role of Nrf2 Expression/Activation	Ref.
B lymphocytes	terminal plasma cell differentiation	Nrf2 target gene expression↑, plasma cell differentiation↓↑	[102]
	Nrf2 WT vs. KO mice, splenocytes-derived B cells, LPS-treated, tBHQ	IgM↑, CD25↓, CD69↓, CD22↓, plasma cell differentiation (CD138) ↓	[103]
	PBMC derived B cells, 15-dPGJ_2_	HO-1↑	[104]
Dendritic cells	PBMC-derived DCs, glioma-dependent microenvironment Nrf2 siRNA	DC maturation and activation↓, restored by Nrf2 knockdown	[105]
	Nrf2 WT vs. knock-out mice, BM-derived DCs	iDC: GSH↑, CD80/CD86↓, phagocytosis↑, antigen presentation↑	[106]
	Nrf2 WT vs. knock-out mice, BM- and myeloid lung derived DCs	pro-inflammatory markers↓	[107]
Granulocytes	siNrf2, TMZ	migration↑	[59]
	Nrf2 WT vs. KO mice,	zymosan-dependent activation↑ and migration↑	[108]
	primary neutrophils	Nrf2 is highly expressed	[109]
	primary neutrophils (PBMCs), LPS treatment RTA-402	pro-inflammatory gene expression↓	[110]
Monocytes/ Macrophages	HIV, SFN	HIV infection↓	[111]
	Nrf2 WT vs. KO mice BM-derived MΦ	antigen-driven CD8+ T cell function↓ by limiting MΦ -dependent GSH and Cys availability	[112]
	endotoxin model [15 mg/kg], LPS treated primary human MΦ derived from PBMCs and primary mouse MΦ BM-derived	itaconate-dependent activation of Nrf2 linking Nrf2 activation to cell metabolism	[113]
	COPD, intranasal application of H. influenza and P. aeruginosa, alveolar MΦ; SFN	MARCO expression↑, bacterial phagocytosis↑	[114]
	BM-derived myeloid lineage specific Keap1 KO (LysM^Cre^/Keap1^fl/fl^) MΦ, M1 vs. M2 polarized, DEM, 15d-PGJ_2_	MΦ M1-induced gene expression ↓ MΦ M2-induced gene expression↓↑	[115]
T lymphocytes	invariant natural killer T cells, CD4^+^ T cell specific Keap1 knock-out mice or Nrf2 knock-out	NKT cell development, homeostasis, and metabolism↓, NKT2 and NKT17↑, NKT1↓, additional Nrf2 knock-out restored all Keap1 mediated effects	[116]
	T_regs_, Foxp3-specific Keap1 KO	T_regs_↓	[117]
	Nrf2 WT mice, experimental autoimmune uveitis, draining lymph nodes, spleen, NaB	Th17↓, T_regs_↑, HO-1↑	[118]
	Th17, mixed granulocyte airway inflammation, sensitization and challenge via cockroach allergen extract, SFN	Th17↓, IL-6↓, IL-17A↓, IL-23↓	[119]
	Nrf2 WT vs. KO mice, CD4^+^ and CD8^+^ T cells, acute graft versus host disease in mice	CD4^+^ T cells↓, CD8^+^ T cells↓↑, nTreg Helios^+^↑	[120]
	CRISPR/Cas-dependent Keap1 knock down in Jurkat T cells and primary human T lymphocytes	CD4^+^ T cells↑, CD8^+^ T cells↓, Nrf2-dependent target genes↑	[97]
	CD4^+^ T cell specific Keap1 knock-out mice, IR-induced AKI	baseline antioxidant gene expression↑, intrarenal CD25^+^Foxp3^+^ Tregs↑, intracellular level of TNFα, IFNγ, and IL-17↓, AKI↓	[121]
	Nrf2 WT vs. knock-out mice, splenocytes, tBHQ, CDDO-Im	TNFα↓, IFNγ↓, IL-2↑, CD25↓↑, CD69↓↑, NF-κB↓, c-Jun↑, Th1↓, Th2↑	[122]
	Nrf2 WT vs. knock-out mice, lymph nodes, spleen, αCD3/CD28-ab treated, tBHQ	IFNγ↓, Nrf2 target genes (NQO1, GCLC, HO-1); Th2 markers (IL-4, IL-5, IL-13)↑	[123]
	human CD4^+^ T cells derived from PBMCs, αCD3/CD28-ab treated, Nrf2 siRNA, tBHQ	Nrf2 target genes (NQO1, GCLM, HO-1, TRX1)↑, activation markers (IL-2, TNFα, IFNγ, IL17)↓↑	[124]

## Abbreviations

15d-PGJ2: 15-deoxy-Δ12,14-prostaglandin J2; ab, antibody; ABTS, 2,2′-azino-bis(3-ethylbenzothiazoline-6-sulfonic acid); Alb, albumin; allo-HCT, allogenic hematopoietic transplantation; ALT, alanine aminotransferase; ARDS, acute respiratory distress syndrome; AKI, acute kidney injury; ALI, acute liver injury; ALI, acute lung injury; ARE, antioxidant-responsive element; Bach1, BTB domain and CNC homolog 1; BARD-Me, bardoxolone methyl; BCR, B-cell receptor; BBS4, Bardet-Biedl syndrome4; ß-TrCP, ß-transducin repeat-containing protein; CAC, citric acid cycle; CAD, coronary artery disease; Cas9, CRISPR-associated 9; CASP, colon ascendens stent peritonitis; CAT, catalase; CD, cluster of differentiation; CDDO-Im, 1-[2-cyano-3-,12-dioxooleana-1,9(11)-dien-28-oyl]imidazole; CDDO-Me, 1-[2-cyano-3-,12-dioxooleana-1,9(11)-dien-28-oyl] methyl ester; CKD, chronic kidney disease; CLP, cecal ligation and puncture; COPD, chronic obstructive pulmonary disease; Cre, causes recombination; CRISPR, clustered regularly interspaced short palindromic repeats; CUL, cullin; CXCR, C-X-C chemokine receptor; DC, dendritic cell; DEM, diethylmaleate; DMF, dimethyl fumarate; DMI, dimethyl itaconate; EAU, experimental autoimmune uveitis; fMLP, N-formyl-methionyl-leucyl-phenylalanine; FoxP3, forkhead box protein P3; GCL, glutamate cysteine ligase; GCLC, glutamate cysteine ligase catalytic subunit; GCLM, glutamate cysteine ligase modifier subunit; GLI2, glioma-associated oncogene 2; GSH, glutathione; GSR, glutathione-reductase; GSSG, glutathione disulfide; GST, glutathione-S-transferase; GvHD, graft versus host disease; Hh, hedgehog; HO-1, heme-oxygenase-1; ICU, intensive care unit; Keap1, Kelch-like ECH-associated protein 1; IFN, interferon; IL, interleukin; IR, ischemia-reperfusion; Irg1, immune-responsive gene 1; LPS, lipopolysaccharide; LysM, lysozyme M; MARCO, macrophage receptor with collagenous structure; MDA, malondialdehyde; MODS, multi organ dysfunction; NAC, N-acetyl cysteine; NAT, arylamine-N-acetyltransferase; NaB, sodium butyrate; NQO1, nicotinamide adenine dinucleotide phosphate (NADPH) quinone oxidoreductase 1; Nrf2, NF-E2 p45-related factor 2; OFD1, oral-facial-digital syndrome 1; OI, 4-octyl itaconate; PAH, pulmonary arterial hypertension; PH, pulmonary hypertension; PKC, protein kinase C; PTCH1, patched 1; PTM, posttranslational modifications; RBX, RING-box protein; ROS, reactive oxygen species; SDH, succinate dehydrogenase; SFN, sulforaphane; SFX-01, sulforadex; SIRS, systemic inflammatory response syndrome; SKP1, S-phase kinase-associated protein-1; sMaf, small muscolo-aponeurotic fibrosarcoma; SnPP, tin protoporphyrin IX; SQSTM1, sequestosome 1; SULT, sulfotransferase; TAC, total antioxidant capacity; tBHQ, tert-butylhydroquinone; TCA, tricarboxylic acid cycle; TLR4, toll-like-receptor 4; TMZ, tert-butylhydroquinone; TNF, tumor necrosis factor; TPA, 12-O-tetradecanoylphorbol-13- acetate; TRX, thioredoxin; TRXR, thioredoxin reductase; TXNRD, thioredoxin reductase; Ubc9, ubiquitin-conjugating enzyme 9; UDP, uridine diphosphate; UGT, UDP glycosylase.
